# Young man with acute pain in the hypogastrium: What is the diagnostic?

**DOI:** 10.1002/ccr3.1734

**Published:** 2018-07-22

**Authors:** Youness Jabbour, Souhail Regragui

**Affiliations:** ^1^ Urology B Department Ibn Sina Teaching Hospital Rabat Morocco; ^2^ Faculty of Medicine and Pharmacy Rabat Morocco

**Keywords:** hypogastrium, lithiasis, pancake kidney, renal ectopy

## Abstract

Congenital renal anomalies can stay asymptomatic even until adult age and be revealed by occurrence of complications. Clinicians should keep in mind this eventuality, especially in the countries where screening for congenital abnormalities is not adopted. Ultrasound in front of abdominal pain is useful and can sometimes rectify the diagnosis.

## 1 CASE PRESENTATION

Suprapubic pain in young man can be caused by prostatitis, bowel disease, urinary retention, and bladder stones. Our case illustrates a particular mode of revelation of a rare congenital renal anomaly.

We report the case of a 31‐year‐old patient presenting to emergency department with the onset of an acute hypogastric pain. He had no particular medical history. Except a pollakuria, no other associated clinical signs were mentioned.

Clinical examination found tenderness in the hypogastric area.

**Figure 1:**
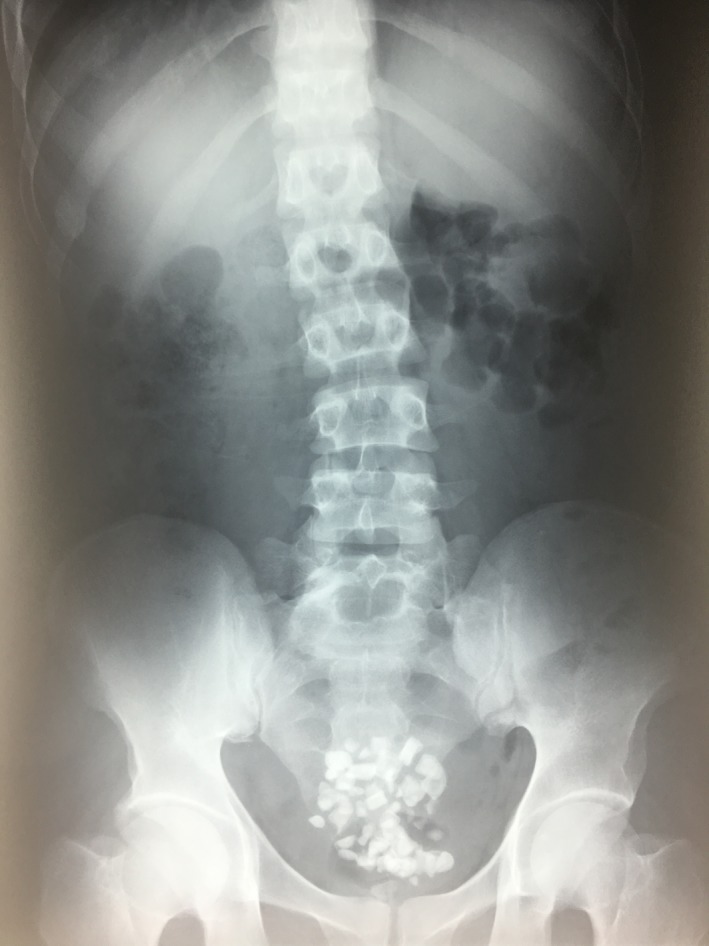
Abdominal X‐ray showing multiple calcifications in suprapubic area

An abdominal X‐ray was performed revealing multiple calcifications in the hypogastrium first thought to be vesical stones (Figure 1). Ultrasonography showed bilateral empty renal fossa.

**Figure 2:**
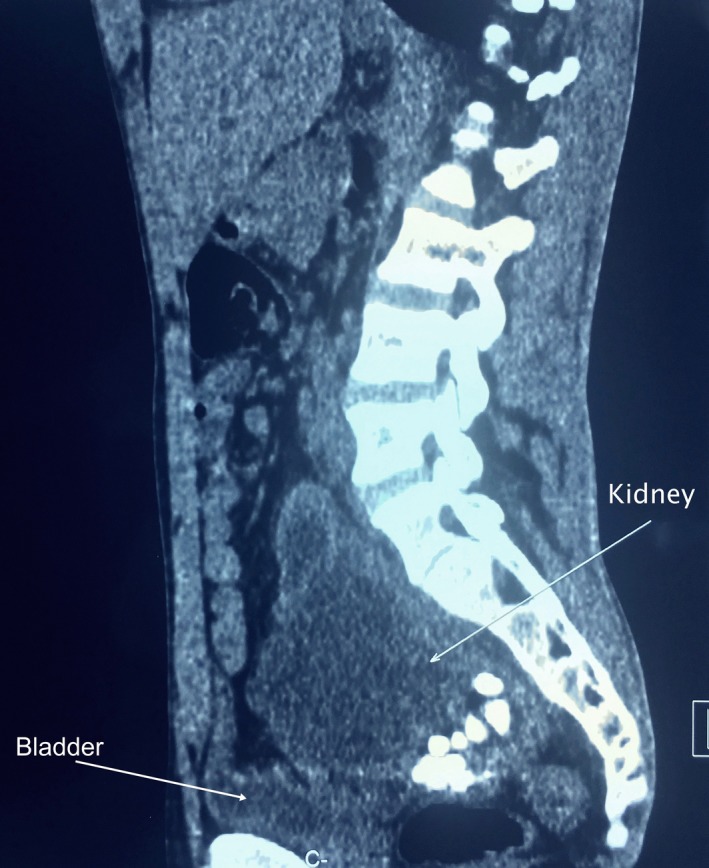
Abdominopelvic CT scan demonstrating a hydronephrotic pelvic kidney with laminated parenchyma and renal stones

Abdominopelvic CT scan confirmed ultrasonography findings and revealed a hydronephrotic fused kidney located in the pelvis with laminated parenchyma and the presence of multiple obstructive renal stones. (Figure 2).

Congenital renal anomalies of position and fusion are the results of impaired cephalic migration from the pelvis to the flank of the ureteric bud and metanephric blastema.^1^

Pelvic kidney is the most common form of ectopic kidney, but its fusion with the contralateral kidney in pelvic position giving a cake kidney is rare and represents only 2% of all fused kidney types.

Cake kidney remains generally asymptomatic, but the close relationship of pelvic kidneys with the surrounding organs explains borrowing symptomatology and diagnostic errors.

This condition may be associated with more serious congenital anomalies that should be evaluated.^2^

## CONFLICT OF INTEREST

None declared.

## AUTHORSHIP

All the authors made a substantial contribution to the preparation of this manuscript and approved the final version for submission. YJ: Designed and drafted the manuscript and procured the clinical images. SR: Performed the literature search.

## INFORMED CONSENT

Informed consent has been obtained for the publication of this clinical image.
